# Variation in cancer surgical outcomes associated with physician and nurse staffing: a retrospective observational study using the Japanese Diagnosis Procedure Combination Database

**DOI:** 10.1186/1472-6963-12-129

**Published:** 2012-05-28

**Authors:** Hideo Yasunaga, Hideki Hashimoto, Hiromasa Horiguchi, Hiroaki Miyata, Shinya Matsuda

**Affiliations:** 1 Department of Health Management and Policy, Graduate School of Medicine, The University of Tokyo, 7-3-1 Hongo, Bunkyo-ku, Tokyo, Japan; 2 Department of Health Economics and Epidemiology Research, School of Public Health, The University of Tokyo, 7-3-1 Hongo, Bunkyo-ku, Tokyo, Japan; 3 Department of Health Quality Assessment, Graduate School of Medicine, The University of Tokyo, 7-3-1 Hongo, Bunkyo-ku, Tokyo, Japan; 4 Department of Preventive Medicine and Community Health, University of Occupational and Environmental Health, Fukuoka, Japan

## Abstract

**Background:**

Little is known about the effects of professional staffing on cancer surgical outcomes. The present study aimed to investigate the association between cancer surgical outcomes and physician/nurse staffing in relation to hospital volume.

**Methods:**

We analyzed 131,394 patients undergoing lung lobectomy, esophagectomy, gastrectomy, colorectal surgery, hepatectomy or pancreatectomy for cancer between July and December, 2007–2008, using the Japanese Diagnosis Procedure Combination database linked to the Survey of Medical Institutions data. Physician-to-bed ratio (PBR) and nurse-to-bed ratio (NBR) were determined for each hospital. Hospital volume was categorized into low, medium and high for each of six cancer surgeries. Failure to rescue (FTR) was defined as a proportion of inhospital deaths among those with postoperative complications. Multi-level logistic regression analysis was performed to examine the association between physician/nurse staffing and FTR, adjusting for patient characteristics and hospital volume.

**Results:**

Overall inhospital mortality was 1.8%, postoperative complication rate was 15.2%, and FTR rate was 11.9%. After adjustment for hospital volume, FTR rate in the group with high PBR (≥19.7 physicians per 100 beds) and high NBR (≥77.0 nurses per 100 beds) was significantly lower than that in the group with low PBR (<19.7) and low NBR (<77.0) (9.2% vs. 14.5%; odds ratio, 0.76; 95% confidence interval, 0.68–0.86; p < 0.001).

**Conclusions:**

Well-staffed hospitals confer a benefit for cancer surgical patients regarding reduced FTR, irrespective of hospital volume. These results suggest that consolidation of surgical centers linked with migration of medical professionals may improve the quality of cancer surgical management.

## Background

Cancer is one of the major causes of death in developed nations, and it is the leading cause of death in Japan [1]. The frequency of cancer surgeries has also been increasing in Japan from 30,605 per month in 1996 to 44,010 per month in 2008 [2], presumably due to population ageing, improved access to cancer screening, and a wider use of surgery because of development of less invasive approaches for previously untreatable patients. With the rise of cancer surgical cases, better allocation of limited healthcare resources is crucial to optimize cancer surgical management and improve operative outcomes.

Numerous studies have reported an association between hospital volume and cancer surgical outcomes in the US [3-5] and Japan [6-9]. Previous studies have also suggested that professional staffing is associated with better short-term outcomes, including physician staffing [10-12] and nurse staffing [13-15]. However, little is known about the concurrent effects of professional staffing and hospital volume on surgical outcomes.

Japan is unique in that the numbers of physician/nurses per bed are extremely low compared with Western standards; there are 26.5 physicians and 117.8 nurses per 100 beds in Japan, while there are 96.1 and 268.1, respectively, in Organization for Economic Cooperation and Development countries [16]. This situation has been created by an excess in the number of hospitals and beds. Regarding nurse staffing, the Japanese government has established standard criteria for the nurse-to-bed ratio in the public health insurance system, which has given hospital administrators a financial incentive to increase the nurse-to-bed ratio. With regard to physician staffing, only the minimum standard (at least 1 physician per 16 acute care beds in Medical Service Law) is set without further incentives to raise the physician-to-bed ratio, which varies widely between hospitals. Under such an extremely low end of staffing, improvement of professional staffing remains an important policy issue in Japan [17].

In the present study, we hypothesized that better staffing of physicians and nurses, even in the extremely low end observed in Japan, is independently associated with better postoperative early outcomes following cancer surgery, irrespective of hospital volume. To prove this hypothesis, we used a national inpatient database in Japan, and performed multivariate analyses to confirm the relation between physician/nurse staffing and operative outcomes, adjusting for hospital volume as well as patient characteristics. Regarding outcome measures, we used the “failure to rescue”—mortality among patients with postoperative complications—because how successfully hospitals rescue patients from surgical complications may be a sensitive indicator for evaluating quality of surgical care [18,19].

A better understanding of the relationship between professional staffing and outcomes may lead to health policy innovation for more efficient resource allocation to increase the benefit to cancer surgical patients. We discuss the implications of our results that could be useful for health policy decision makers in any country.

## Methods

### Data source

We used the Diagnosis Procedure Combination (DPC) database and the Survey of Medical Institutions data. The DPC is a case-mix patient classification system, launched in 2002 by the Ministry of Health, Labour and Welfare of Japan, and is linked with a lump-sum per-diem payment system. All the 82 university hospitals are obliged to adopt the DPC system, but participation by other community hospitals, private or public, is voluntary. Participating hospitals included 855 in 2008, covering approximately 2.9 million inpatients, or approximately 40% of all acute care inpatient hospitalizations in Japan. For this study, we used the data of 2007 and 2008 that included 5.85 million discharge cases.

The DPC system mandates participating hospitals to have electronic submission of claim bills and some clinical data of all the patients discharged between July 1 and December 31 each year, and a copy of the submitted data was collected for research purposes by the research group. The database includes the following: patients’ age and sex; main diagnoses, pre-existing comorbidities, and post-admission complications coded by the International Classification of Disease and Related Health Problems, 10th Revision (ICD-10) codes; and surgical procedures coded by the Japanese original surgical coding system, which is comparable with the ICD, 9th Revision, Clinical Modification (ICD-9-CM) codes. The data also include discharge status [20,21]. In the DPC database, complications that occurred after admission are clearly differentiated from comorbidities that were already present at admission. To optimize the accuracy of the recorded diagnoses, physicians in charge are obliged to record the diagnoses with reference to medical charts.

The Survey of Medical Institutions is a census of hospitals in Japan, conducted every 3 years. The survey data contains structural information such as the number of beds, the number of full-time employed physicians, and the number of nurses in full-time equivalent. We linked the data to the DPC database using hospital identifiers as a linkage key. Because of the anonymous nature of the data, the requirement for informed consent was waived. Study approval was obtained from the Institutional Review Board in the University of Occupational and Environmental Health.

### Patient selection

We identified patients who had undergone elective cancer surgery including (i) lung lobectomy for lung cancer (excluding pneumonectomy), (ii) esophagectomy for esophageal cancer, (iii) gastrectomy for gastric cancer, (iv) colorectal cancer surgery (including colectomy for colon cancer and anterior resection or abdominoperineal resection for rectal cancer), (v) hepatectomy for hepatic cancer, or (vi) pancreatectomy for pancreatic cancer. These six surgeries are major oncological surgeries, which generally have a higher operative mortality than other procedures in general and thoracic surgery [3-5]. Those who underwent two or more cancer surgeries during one hospitalization were excluded.

Preoperative comorbidities included diabetes mellitus (ICD 10 codes, E10-E14), hypertension (I10-I15), cardiac diseases (I20-I25, ischemic heart diseases; I30-I52, other forms of heart diseases), cerebrovascular disease (I60-I69), chronic lung diseases (J40-J47), liver cirrhosis (K74), and chronic renal failure (N18). Based on Quan’s protocol [22], each ICD-10 code of comorbidity was converted into a score, and was summed up for each patient to calculate a Charlson Comorbidity Index (CCI).

### Professional staffing and hospital volume

In Japan, there are two types of nursing licenses, including a registered nurse and practical nurse, but there is no mid-level provider’s license, such as a physician assistant or nurse practitioner. Using the Survey of Medical Institutions data, we estimated the number of physicians per 100 beds (physician-to-bed ratio, PBR) and the number of nurses per 100 beds (nurse-to-bed ratio, NBR) for each hospital. Our data included the number of all the full-time employed physicians, including residents and attending physicians. The number of nurses included the full-time equivalent numbers of all the licensed nurses, but did not include the number of non-licensed providers, such as nurse aids. PBR and NBR are considered to be correlated and the problem of multicollinearity could occur if these two continuous variables were included in a multivariate model. To avoid this problem, PBR and NBR were combined into a single categorical variable including the following four groups: (i) Group A (*below* median PBR and *below* median NBR), (ii) Group B (*below* median PBR and *above* median NBR), (iii) Group C (*above* median PBR and *below* median NBR), and (iv) Group D (*above* median PBR and *above* median NBR).

Hospital volume was defined as the number of each surgical procedure performed annually at each hospital, and was categorized into tertiles (low-, medium-, and high-volume), with approximately equal numbers of patients in each group.

### Outcomes

The outcome measurements included postoperative complications, inhospital mortality and failure to rescue (FTR). Postoperative complications included surgical site infection (T793, T814), peritonitis (K65), sepsis (A40, A41), respiratory complications (pneumonia [J12-J18], postprocedural respiratory disorders [J95] or respiratory failure [J96]), pulmonary embolism (I26), cardiac events (acute coronary events [I21-I24] or heart failure [I50]), stroke (cerebral infarction or hemorrhage [I60-I64]), and acute renal failure (N17).

FTR was defined as the proportion of inhospital death cases among those who had experienced a postoperative complication [18,19]. Therefore, FTR identifies whether the patient is successfully rescued from the complication. An underlying assumption of the FTR theory is that complications reflect patient severity, and the rescue of patients with complications depends on quick identification and aggressive treatment of complications [18,19]. There is ongoing controversy on how FTR should be calculated, because previous FTR studies have used different sets of complications. Silber’s original FTR used a comprehensive set of complications, but several modified FTRs have used limited definitions. For example, a “nurse sensitive” definition only included six complications (pneumonia, shock, gastrointestinal bleeding, cardiac arrest, sepsis and deep venous thrombosis) [18]. Our original set of complications comprised common complications in general and thoracic surgery. We excluded rare complications in general and thoracic surgery, which were involved in Silber’s definition, such as gangrene, amputation, decubitus ulcers, orthopedic complications and compartment syndromes.

### Data analyses

Patient characteristics were summarized by four categories of physician/nurse staffing. We performed univariate comparisons of explanatory variables using a χ^2^ test or an analysis of variance as appropriate. Inhospital mortality, postoperative complication rates, and FTRs were compared across physician/nurse staffing categories. Multivariate analyses were then performed to model the concurrent effects of potentially influential factors (age, sex, CCI, hospital volume, and physician/nurse staffing) on the outcomes using multi-level logistic regression analyses. Data were structured hierarchically into two levels: hospitals and patients. We accounted for clustering of outcomes within hospitals using mixed effects models. This approach is commonly used instead of basic regression approaches because outcomes of patients in the same hospital may be correlated, thus violating independence assumptions made by traditional regression procedures [23,24]. The threshold for significance was a p value <0.05. All statistical analyses were conducted using SAS ver. 9.2 (SAS Institute, Cary, NC, US).

## Results

A total of 131,394 eligible patients were identified. Hospital volume categories (low, medium and high) were determined to be ≤51, 52–106, and ≥107 per year for lung lobectomy (n = 21,639); ≤9, 10–26, and ≥27 for esophagectomy (n = 3,917); ≤47, 48–93, and ≥94 for gastrectomy (n = 35,978); ≤66, 67–119, and ≥120 for colorectal surgery (n = 51,878); ≤22, 23–58, and ≥59 for hepatectomy (n = 10,921); and ≤13, 14–29, and ≥30 for pancreatectomy (n = 7,061).

Table 1 shows that the proportions of patients in the low-, medium- and high-volume groups were almost equal (33.6%, 33.0% and 33.4%, respectively). Lower volume hospitals were more likely to have a lower PBR and NBR. The median PBR was 19.7 (interquartile range, 14.6–27.3) per 100 beds and the median NBR was 77.0 (68.2–86.1) per 100 beds. These numbers were used as cutoff points to categorize physician/nurse staffing into four categories. The mean age was highest in Group A. Patients in Groups C and D had higher rates of several comorbidities. Consequently, CCI was higher among patients in Groups C and D than in those in Groups A and B.

**Table 1:** Patient characteristics

	Total	Group A: low PBR, low NBR	Group B: low PBR, high NBR	Group C: high PBR, low NBR	Group D: high PBR, high NBR	p
Number of patients	131,394	44,758	21,705	22,837	42,094	
Age (average ± SD, years)	67.8 ± 11.5	69.0 ± 11.0	68.4 ± 11.2	66.5 ± 11.8	66.8 ± 11.7	<0.001
Sex (males,%)	62.8	62.4	62.1	62.9	63.5	0.001
Preoperative comorbidities (%)						
Hypertension	17.5	16.2	15.9	19.2	18.7	<0.001
Diabetes mellitus	13.6	13.1	12.6	14.4	14.3	<0.001
Cardiovascular diseases	94.0	94.3	94.7	94.0	93.1	<0.001
Chronic lung diseases	4.9	4.1	3.9	5.2	6.1	<0.001
Liver cirrhosis	1.6	1.2	1.3	2.0	1.8	<0.001
Chronic renal failure	0.70	0.71	0.58	0.80	0.71	0.055
Cerebrovascular diseases	0.48	0.55	0.43	0.43	0.47	0.061
Charlson Comorbidity Index (%)						
0-2	61.2	64.3	63.4	59.2	57.8	<0.001
3-5	26.6	24.2	24.6	27.9	29.5	
6-	12.2	11.5	12.0	12.9	12.7	
Hospital volume						
Low	33.6%	58.2%	37.0%	16.3%	15.1%	<0.001
Medium	33.0%	27.0%	37.4%	35.1%	36.0%	
High	33.4%	14.8%	25.6%	48.6%	49.0%	

PBR, physician-to bed ratio (low, <19.7 physicians per 100 beds; high, ≥19.7); NBR, nurse-to-bed ratio (low, <77.0 nurses per 100 beds; high, ≥77.0).

Overall, postoperative complications were observed among 3.8% of patients for surgical site infection, 3.1% for sepsis, 3.1% for respiratory complications, 2.6% for peritonitis, 1.9% for cardiac events, 1.0% for acute renal failure, 0.86% for stroke, and 0.20% for pulmonary embolism. In total, 15.2% of all patients had at least one complication. Overall inhospital mortality was 1.8% and the FTR rate was 11.9%.

**Figure 1:**
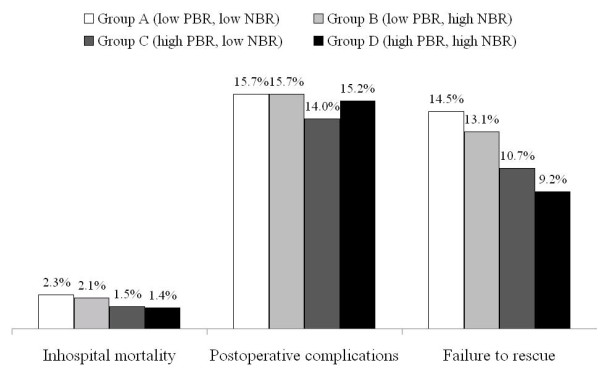
**Relationship between physician/nurse staffing and cancer surgical outcomes.** PBR, physician-to bed ratio (low, <19.7 physicians per 100 beds; high, ≥19.7); NBR, nurse-to-bed ratio (low, <77.0 nurses per 100 beds; high, ≥77.0).

Figure 1 illustrates the rates of inhospital mortality, postoperative complications, and FTR by the four categories of physician/nurse staffing. Patients with a higher PBR showed lower morality, complication rates, and FTR rates.

Table 2 shows the results of logistic regression analysis for FTR. Even after adjustment for patients’ conditions and hospital volume, FTR rates were significantly different between Groups A and D (odds ratio, 0.76 [95% confidence interval, 0.63–0.90]; p = 0.002), but not between Groups A and B (0.94 [0.78–1.13]; p = 0.505) or between Groups A and C (0.91 [0.73–1.13]; p = 0.379). Group D showed a relatively lower FTR rate than Group C, but this was not significant (0.83 [0.66–1.05]; p = 0.128).

**Table 2:** Logistic regression analysis for failure to rescue

	odds ratio	95% confidence interval			p
Age (10-year age increase)	1.50	1.43	-	1.57	<0.001
Sex (Female vs. male)	0.79	0.72	-	0.88	<0.001
Charlson Comorbidity Index	1.03	1.01	-	1.05	0.002
Hospital volume					
Low	1.00				
Medium	0.89	0.79	-	1.01	0.077
High	0.62	0.53	-	0.73	<0.001
Physician and nurse staffing					
Group A (low PBR, low NBR)	1.00				
Group B (low PBR, high NBR)	0.94	0.78	-	1.13	0.505
Group C (high PBR, low NBR)	0.91	0.73	-	1.13	0.379
Group D (high PBR, high NBR)	0.76	0.63	-	0.90	0.002

PBR, physician-to bed ratio (low, <19.7 physicians per 100 beds; high, ≥19.7); NBR, nurse-to-bed ratio (low, <77.0 nurses per 100 beds; high, ≥77.0).

When we conducted a similar analysis on inhospital mortality, Group D showed a significantly lower mortality compared with Group A (0.82 [0.71–0.95]; p = 0.009), while postoperative complication rates were not different among the groups (1.01 [0.90–1.13]; p = 0.918 for Group D vs. Group A).

Table 3 shows the results of post-hoc analyses of FTR rates in the four physician/nurse staffing groups by the types of surgery. FTR was significantly related to physician/nurse staffing in lung lobectomy, esophagectomy, gastrectomy, colorectal surgery, and pancreatectomy.

**Table 3:** Failure to rescue in the four physician/nurse staffing groups for each surgery

				FTR (%)					
	N	Inhospital mortality (%)	Postoperative complications(%)	Total	Group A: low PBR, low NBR	Group B: low PBR, high NBR	Group C: high PBR, low NBR	Group D: high PBR, high NBR	p
Lung lobectomy	21,639	0.92	10.2	9.0	15.3	12.9	7.9	5.9	<0.001
Esophagectomy	3,917	4.14	26.3	15.7	21.8	18.7	10.9	13.8	0.001
Gastrectomy	35,978	1.43	13.1	10.9	13.8	10.9	10.7	7.3	<0.001
Colorectal surgery	51,878	2.06	15.8	13.0	14.2	14.3	12.2	10.6	<0.001
Hepatectomy	10,921	2.49	17.4	14.3	17.3	14.3	11.8	14.0	0.061
Pancreatectomy	7,061	2.48	27.8	8.9	12.5	9.0	7.6	6.6	0.001

PBR, physician-to bed ratio (low, <19.7 physicians per 100 beds; high, ≥19.7); NBR, nurse-to-bed ratio (low, <77.0 nurses per 100 beds; high, ≥77.0).

## Discussion

The present study examined the association between cancer surgical outcomes and physician/nurse staffing in relation to hospital volume, using a nationwide administrative database. After adjustment for hospital volume, the FTR rate in the high-PBR-high-NBR group was significantly lower than that in the low-PBR-low-NBR group.

The inverse relationship between better professional staffing and hospital mortality in the present study is consistent with findings in previous studies [10-16]. Few studies have taken into account both professional staffing and hospital volume to evaluate surgical outcomes [13]. Our study revealed that better physician and nurse staffing were independently associated with a lower FTR in general and thoracic cancer surgery, irrespective of hospital volume.

Previously reported volume-outcome relationships may be partly explained by professional staffing. In this context, recent debate on hospital volume as an indicator of quality of care needs careful reconsideration in terms of allocation of a suitable number of qualified physicians and nurses as a structural basis for quality of care.

Volume-outcome relationships have mainly been explained by the “practice-makes-perfect” theory, and case accumulation has been enhanced based on this theory. In fact, growing interest in these relationships has bolstered relevant policy changes, including migration of cancer surgery to high-volume hospitals [25,26]. However, there is ongoing controversy regarding such policy; if patients are directed to higher volume institutions, the increased volume will overwhelm the resources of such institutions, thereby rendering these procedures even less accessible [27].

In accordance with our results, case accumulation should be accompanied by a suitable increase in medical staff. Concentration of physicians and nurses is considered necessary for hospitals regardless of size and case volume.

Efficient resource allocation for improving cancer surgical management is a common healthcare policy issue in any advanced nation. Japan is facing a super-aged society and weakened economy, which threatens the sustainability of the publish health insurance system. Physician shortage is an unsolved problem in Japan; the number of surgeons (including general and thoracic surgeons) is gradually decreasing from 28,425 in 1996 to 26,995 in 2008 [28]. Geographically, 2,522 surgical centers are distributed to an inhabited area of 121,000 km^2^ in Japan (2.1 centers/100 km^2^), as of 2008. A total of 44,010 cancer surgeries were performed in September 2008, and the mean number of cancer surgeries was calculated to be only 17.5 per hospital per month [2]. Therefore, healthcare resource allocation regarding cancer surgery in Japan is characterized as a large number of small hospitals with low case volume.

Based on our results, we speculate that consolidation of surgical centers and simultaneous reallocation of human resources could lead to better outcomes after cancer surgery, particularly in general and thoracic surgery. Migration of medical professions to high volume hospitals is considered essential. This approach should be implemented through the shutdown of low-volume surgical units, even if it will result in increased travel distance for cancer patients.

The Japanese Association of Thoracic Surgery has already initiated an attempt for regionalization of cardiac surgery by restricting its certification criteria for training institutions in 2005. This restriction has required several certified centers in the same regions to consolidate, resulting in an improvement in outcome and a slight decrease in accessibility to cardiac surgeries [29]. Unlike cardiac surgery, most cancer surgeries are elective; therefore, increased patient travel distance for cancer surgery could have less negative effect on health outcomes. Therefore, consolidation of cancer surgical centers may lead to improvement of outcomes that could compensate for a decreased accessibility to surgical care.

Several limitations should be acknowledged. First, we used the number of physicians per bed as an indicator for intensity of physician services, but further knowledge of individual physician characteristics, such as surgeon volume and training status (residents/fellows/board-certified physicians), and nurse characteristics, such as nurse education and the nurse work environment [14] could refine our approach. Second, other important outcomes including recurrence, long-term survival, and subsequent health resource consumption were not investigated in the present study because of data availability. Third, hospitals in the DPC database are not representative of all hospitals in Japan. Specifically, a low participation rate of very small hospitals in the DPC system skews the population being evaluated, and this might have resulted in underestimation of overall mortality. Fourth, the DPC database is an administrative claim database, and recorded diagnoses in such databases are less well validated than those in planned prospective cohorts or registries. Postoperative complications might have been underestimated due to underreporting. Because the DPC database includes only inpatient data, 30-day mortality was not available. Lastly, due to a novel author-derived definition of FTR, results may not compare directly with previously-published work.

## Conclusion

Well-staffed hospitals confer a benefit for patients in terms of reduced FTR. Our results suggest that consolidation of surgical centers together with a concentrated allocation of medical professionals may improve the quality of surgical care for cancer.

## Competing interests

The authors have no competing interests.

## Authors’ contributions

HY and HH1 conceived the study concept and study design. HY and HH2 performed compilation and synthesis of the data. HY and HM carried out statistical analyses. SM supervised the DPC research project. All authors participated in interpretation of the results and writing of the report, and approved the final version.

## Pre-publication history

The pre-publication history for this paper can be accessed here:

http://www.biomedcentral.com/1472-6963/12/129/prepub
